# Preparation of geranium oil formulations effective for control of phenotypic resistant cattle tick *Rhipicephalus annulatus*

**DOI:** 10.1038/s41598-022-14661-5

**Published:** 2022-07-08

**Authors:** Samar M. Ibrahium, Shawky M. Aboelhadid, Ahmed A. Wahba, Ahmed A. Farghali, Robert J. Miller, Abdel-Azeem S. Abdel-Baki, Saleh Al-Quraishy

**Affiliations:** 1 Department of Parasitology, Animal Health Research Institute, Fayum Branch, Fayum, Egypt; 2 Department of Parasitology, Faculty of Veterinary Medicine, Beni-Suef University, Beni-Suef, 52611 Egypt; 3 Department of Parasitology, Animal Health Research Institute, Dokki, Egypt; 4 Materials Science and Nanotechnology Department, Faculty of Postgraduate Studies for Advanced Sciences, Beni-Suef University, Beni-Suef, Egypt; 5 Office of National Programs, United States Department of Agriculture Agricultural Research Service, Washington, USA; 6 Zoology Department, Faculty of Science, Beni-Suef University, Beni-Suef, Egypt; 7 Zoology Department, College of Science, King Saud University, Riyadh, Saudi Arabia

**Keywords:** Animal biotechnology, Nanobiotechnology

## Abstract

The aim of the present study was to evaluate in vitro and in vivo the acaricidal activity of two forms of geranium (*Pelargonium graveolens*) (PG). These two forms were the *P. graveolens* essential oil nanoemulsion (PGN), and the PG in combination with the sesame oil (SO), PGSO). These forms were first evaluated in vitro for their adulticidal, ovicidal, and larvicidal activities against the different stages of acaricide-resistant *Rhipicephalus annulatus* (Say). Geranium nanoemulsion was prepared and then characterized by UV–Vis spectrophotometer, and zeta droplet size measurement. The results revealed that LC_50_ of the PG against the adult ticks was attained at concentration of 7.53% while it was decreased to 1.91% and 5.60% for PGSO and PGN, respectively. Also, the LC_50_ of PGN and PGSO were reached at concentrations of 1.688 and 0.944%, respectively against the larvae while the LC_50_ of the PG was reached at concentration of 3.435% for. The combination of PGN with PG exhibited non-significant ovicidal effect meanwhile PGSO showed significant ovicidal effect even at the low concentration (2.5%). The PGSO and PGN formulations were applied in a field trial to control the ticks of the naturally infested cattle. PGSO and PGN significantly reduced the tick burden to 74.83% and 87.97%, respectively at 3 weeks post-application with performance better than the deltamethrin (29.88%). In conclusion, the two PG forms can be used as suitable alternatives to control *R. annulatus* tick and they need further modifications for effective field application.

## Introduction

*Rhipicephalus annulatus* (Say), the cattle fever tick, is a parasite with economic significance for bovid in tropical and subtropical countries^1^. This tick species induces significant impacts to the production and health of the animals through sucking of the blood, decreasing the body weight gain, and transmitting of the blood-borne protozoa such as *Babesia* spp. and *Anaplasma*^2^. The common method for the tick control is the application of chemical acaricides to infested host animals. Continuous acaricide applications results in significant environmental and public health hazards in addition to the development of acaricide resistant strains. Actually, the tick resistance to deltamethrin and ivermectin has been proven within the area of the present study^1,3,4^.

Essential oils are safe for animals, humans, and the environment. Additionally, there is currently low incidence of resistance in cattle fever ticks to these oils^5^. Therefore, essential oils and their metabolites could be used as an effective alternatives to the chemical acaricide for insects and ticks control^6^. *Pelargonium graveolens* L. is an aromatic medicinal plant belonging to the family Geraniaceae. Its leaves are used as a flavoring, in perfume, insect repellent, and also in aromatherapy for the treatment of gastrointestinal diseases, throat infections, bleeding, anti-inflammatory, diuretic, antiseptic, antidepressant, calmative, and balancing for the endocrine system^7^. Egypt is a major producer for *Pelargonium graveolens*^8^. Also, *P. graveolens* is known for its ability to reduce the reproductive index of the tropical cattle tick (*Rhipicephalus microplus*) with in vitro treatment efficacy reached to 97%^9^. *Pelargonium roseum* is also known to have the ability to decrease the egg mass of treated *Rhipicephalus (Boophilus) annulatus* in Iran^10^. In addition, geranium oil was proven to have an insecticidal effect against stored crop insects including *Sitophilus oryzae*, *Tribolium castaneum* (Herbst) and *Rhyzopertha dominica*, and against larvae of *Culex quinquefasciatus*^11,12^.

The insecticidal activity of *P. graveolens* needs improvement which can be accomplished by the preparation of a nanoemulsion from the essential oils. Nanoemulsions are more stable and can increase the biological activity by decreasing droplet size^13^. Recently, nanocapsules and nanoemulsions containing cinnamon oil showed high efficacy against engorged female of *R. *(*B.*)* microplus*^14,15^. Also, Boito et al.^16^ reported that nanoparticles containing oil of the tea tree interfered in the reproduction of *R. *(*B.*)* microplus*.

Vegetable oils have been shown to be synergistic when combined with essential oils. Sesame oil had a synergistic effect when combined with clove essential oil against the pulse beetle, *Callosobruchus maculatus* adults^17^. Also, Devaramane et al.^18^ found that sesame oil exhibited a high level of suppression in *Sitophilus oryzae* with a suppression rate of 74.48%.

Consequently, the acaricide activity of *P. graveolens* against different stages of *R. *(*B.*)* annulatus* was studied using nanoemulsion and synergism by the addition of sesame and linseed oils. Additionally, a controlled field trial was completed using these forms on naturally infested cattle.

## Results

### GC-Mas analysis of PG

GC-Mas revealed that the main constituents of PG were Citronellol (14.44%), Geraniol (11.08%), Linalool (7.74%), Citronellyl formate (7.66%), 10-epi-y-eudesmol (4.93%), Isomethane (4.35%) and Geranyl formate (3.91%). Table 1 shows the retention time (RT) and the amount (% Area) of the components.

**Table 1:** GC-Mas of *Pelargonium graveolens* essential oil (granium).

RT	Compound name	Area%
3.30	β-Pinene	0.75
3.89	*cis*-Linalool oxide	0.55
4.32	Linalool	7.74
4.72	Rose oxide	0.76
5.17	*Trans*-p-menthone	0.33
5.41	Isomethane	4.35
5.73	Citronellal	0.44
5.85	Isopulegol	0.41
6.63	Citronellol	14.44
6.94	β-Geranial	0.40
7.31	Geraniol	11.08
7.55	Citronellyl formate; formic acid	7.66
8.01	Geranyl formate	3.91
8.69	Geranyl acetal	0.63
8.95	Citronellyl acetate	1.23
9.44	Copaene	1.05
9.63	a-Bourbonene	3.28
10.10	ι-Gurjunene	0.26
10.32	Caryophyllene	2.55
10.49	β-Copaene-4α-ol	0.36
10.75	Aromadendrene	1.73
10.88	ç-Muurolene	0.96
10.99	Humulene	0.83
11.13	Epi-β-Caryophyllene	0.48
11.38	Geranyl propionate	2.01
11.56	Germacrene D	2.63
11.65	Epi-β-Selinene	0.30
11.85	Elemene	1.60
11.91	Epicubebol	0.53
12.04	a-Farnesene	0.30
12.19	Naphthalene	0.89
12.39	σ-Cadinene	3.27
12.64	α-Gurjunene	0.30
12.82	γ-Costol	0.82
13.08	Geranyl butyrate	2.36
13.48	Spathulenol	1.13
13.60	Phenylethyl tiglate	2.71
13.76	(−)-Globulol	0.29
13.91	Neryl 2-methylbutyrate	0.41
14.03	Caryophylene oxide	0.30
14.34	10-Epi-y-eudesmol	4.93
14.42	Cubenol	0.17
14.57	Agarospirol	0.40
14.72	Guaiene	0.69
14.89	Elemol	1.93
15.07	Citronellyl tiglate	0.79
15.40	2,6-Octadiene,2,6-dimethyl	0.47
15.75	Geranyl tiglate	2.62
16.02	Geranyl palmitate	0.84
16.66	Geranyl isobutyrate	0.37
17.77	Citronellol heptanoate	0.23
18.36	Geranyl heptanoate	0.36
20.00	Geranyl caprylate	0.20
	Total	100

### Characterization of PG nanoemulsion

UV–Vis spectrophotometer detected the absorbance peak of PGN at 345 nm (Supplementary Fig. 1). Zeta potential measurements revealed the PG nanoemulsion with negative charges on the outer surface of the oil (− 0.569 mV), hydrodynamic particle size (mean droplet size of around = 17.84 d, nm) and PDI = 0.299. The low value of PDI reflected the homogeneous size distribution of PG droplets (Supplementary Fig. 2, 3).

### Adulticidal activity of PG formulations against R. annulatus tick

The PG showed acaricidal effects in a dose dependent manner. The highest concentrations of 10 and 5% showed high significant mortality (P < 0.05) to ticks at rate of 73.3 and 23.3%, respectively. Meanwhile the lowest concentrations of 2.5, 1.25 and 0.625% did not cause any significant mortality (P > 0.05). The PGN revealed significant mortality (P < 0.05) higher than that of the PG especially at concentration of 10% (96.60% vs. 73.30%, respectively) (Table 2). Although the mortality rate of SO was lower than that of PG, it was still have significant mortality even at the low concentrations (2.5, 1.25 and 0.625%) (Table 2). Linseed oil (LS) did not cause any mortality in adult ticks even at the highest concentrations. Egg production index (EPI) of ticks revealed significant reduction in the egg production of PGSO treated group even at the low concentrations. PG and PGN treated groups showed also significant reduction in the egg production at concentrations of 10 and 5%. Similarly, SO treated group showed reduction in the egg production which comparably lower than that of PGSO treated one. LS showed EPI close to that of the negative control group (Table 3). PGSO was prepared at different ratios to determine the most effective combination. 9PG:1SO, 8PG:2SO, 5PG:5SO and 1PG:9SO were the most effective ratios (Table 4). The LC_50_ of PG and PGN were reached at concentrations of 7.53, and 5.60%, respectively. The combination of PGSO showed a synergistic effect with a significant synergistic factor of 3.94, and its LC_50_ was attained at concentration lower than that required for the LC_50_ of PG alone (1.91% vs. 7.53%, respectively). On contrary, the PGLS combination showed an antagonistic effect with synergistic factor less than 1.00 (Table 5). Moreover, the deltamethrin control treated group (1 μl/ml) showed results similar to that of 70% ethyl alcohol control treated one. Meanwhile, the control positive group that treated with Phoxim revealed 100% adult mortality.

**Table 2:** Adulticidal activity of tested EOs on *R. annulatus* (the ratio between oils binary mixture 1:1).

Concentrations%	10	5	2.50	1.25	0.625
PG (DF%)	73.33 ± 0.577*	23.33 ± 0.577*	10.00 ± 1.732	6.660 ± 1.154	0.000 ± 0.000
PGN (DF%)	96.66 ± 0.577*	30.00 ± 0.000*	20.00 ± 0.000*	16.66 ± 1.154*	3.330 ± 0.577
SO (DF%)	66.66 ± 0.577*	56.66 ± 0.577*	40.00 ± 1.000*	26.66 ± 0.577*	16.66 ± 0.577*
LS (DF%)	16.66 ± 0.577	13.33 ± 0.577	6.660 ± 0.577	0.000 ± 0.000	0.000 ± 0.000
PGSO (DF%)	100.0 ± 0.000*	100.0 ± 0.000*	53.33 ± 0.577*	46.66 ± 0.577*	23.33 ± 0.577*
PGLS (DF%)	16.66 ± 0.577	6.660 ± 0.577	3.330 ± 0.577	0.000 ± 0.000	0.000 ± 0.000
Control (deltamethrin 1, 2, 3 and 4 μl/1 ml)			0.000 ± 0.000		
Control (phoxim 0.50 μl/1 ml)			100.0 ± 0.000		
Control (ethyl alcohol 70%)			0.000 ± 0.000		

*DF%* female mortality%, *PG* *Pelargonium graveolens* oil, *PGN* *Pelargonium graveolens* nanoemulsion, *SO* sesame oil, *LS* linseed oil, *PGSO* *Pelargonium graveolens* oil + sesame oil, *PGLS* *Pelargonium graveolens* oil + linseed oil.

(*)Significant to control (ethyl alcohol) (P < 0.05).

**Table 3:** Egg production index (EPI) of EOs treated adult *R. *(*B*.)* annulatus* (the ratio between oils binary mixture 1:1).

Concentrations%	10	5	2.50	1.25	0.625
PG (EPI%)	21.54 ± 1.547*	26.52 ± 0.6377*	47.15 ± 2.892	50.57 ± 1.250	56.31 ± 3.026
PGN (EPI%)	3.029 ± 0.223*	22.18 ± 1.736 *	34.54 ± 2.561 *	41.87 ± 0.997	54.28 ± 1.060
SO (EPI%)	22.00 ± 0.455*	25.32 ± 1.027 *	27.03 ± 1.027 *	32.10 ± 0.075 *	35.19 ± 3.157 *
LS (EPI%)	44.90 ± 0.942	47.70 ± 2.213	50.37 ± 1.832	51.89 ± 1.690	51.30 ± 4.834
PGSO (EPI%)	0.000 ± 0.000*	0.000 ± 0.000*	13.58 ± 2.475 *	16.30 ± 0.234 *	24.03 ± 1.487 *
PGLS (EPI%)	45.04 ± 3.744	48.03 ± 3.603	49.15 ± 4.962	49.93 ± 1.445	55.21 ± 1.990
Control (deltamethrin 1, 2, 3 and 4 μl/1 ml)			49.34 ± 7.987		
Control (phoxim 0.50 μl/1 ml)			0.000 ± 0.000		
Control (ethyl alcohol 70%)			55.15 ± 1.560		

*PG* *Pelargonium graveolens* oil, *PGN* *Pelargonium graveolens* nanoemulsion, *SO* sesame oil, *LS* linseed oil, *PGSO* *Pelargonium graveolens* oil + sesame oil, *PGLS* *Pelargonium graveolens* oil + linseed oli, *EPI* egg production index%.

(*)Significant to control (Ethyl alcohol) (P < 0.05).

**Table 4:** Adulticidal activity of PGSO in different ratios.

PGSO (%)	Mean ± standard deviation
10 SO	66.66 ± 0.577
1PG + 9SO	90.00 ± 1.000
2PG + 8SO	60.00 ± 0.000
3PG + 7SO	50.00 ± 0.000
4PG + 6SO	76.66 ± 1.154
5PG + 5SO	90.0 ± 0.000
6PG + 4SO	73.33 ± 1.527
7PG + 3SO	86.66 ± 1.154
8PG + 2SO	93.33 ± 0.577
9PG + 1SO	100.0 ± 0.000
10 PG	73.33 ± 0.577
Control (deltamethrin 1 μl/1 ml)	00.00 ± 0.000
Control (phoxim 0.50 μl/1 ml)	100.0 ± 0.000
Control (ethyl alcohol 70%)	00.00 ± 0.000

**Table 5:** LC_50_, LC_90_ and synergistic factor of adulticidal activity of PG against *R.*(*B.*)* annulatus* (the ratio between oils in the binary mixture 1:1).

Oil	LC_50_ %	LC_90_ %	Synergistic factor
PG	7.53	12.1	
PGN	5.60 *	9.47	
SO	5.86	13.4	
LS	15.78	25.0	
PGSO	1.91 *	3.68	3.94 (synergism)
PGLS	15.45	23.2	0.48 (antagonism)

*PG* *Pelargonium graveolens* oil, *PGN* *Pelargonium graveolens* nanoemulsion, *SO* sesame oil, *LS* linseed oil, *PGSO* *Pelargonium graveolens* oil + sesame oil, *PGLS* *Pelargonium graveolens* oil + linseed oil.

### Ovicidal effect of PG formulations

Application of the PG on the eggs of ticks induced a significant increase in the egg mortality (P < 0.05) with unhatching rates of 99.0 and 51.3% at the concentrations of 10 and 5%, respectively. Regarding the SO, only the concentration of 10% caused significant unhatching rateof 58.33%. All eggs hatched normally in the LS treated groups at all the used concentrations. The PGSO combination showed a synergistic effect on the total eggs produced by the ticks at the different used ratios (Table 6). The PGN and the PG showed the same effect on the eggs hatchability with no significant difference between both of them.

**Table 6:** Ovicidal effect of Eos (the ratio between oils in the binary mixture 1:1).

Concentrations%	10	5	2.50	1.25	0.625
PG (unhatched egg% mean ± st. deviation)	99.00 ± 1.000*	51.33 ± 3.214 *	5.000 ± 1.000	2.000 ± 1.000	0.666 ± 0.577
PGN (unhatched egg% mean ± st. deviation)	99.33 ± 1.000*	52.33 ± 2.214 *	5.660 ± 1.000	2.330 ± 1.000	0.666 ± 0.577
SO (unhatched egg% mean ± st. deviation)	58.33 ± 1.527*	5.000 ± 1.000	2.000 ± 1.000	2.000 ± 1.000	1.000 ± 1.000
PGSO (unhatched egg% mean ± st. deviation)	99.00 ± 1.000*	89.33 ± 1.154 *	67.66 ± 2.516 *	5.000 ± 1.000	0.666 ± 0.577
LS (unhatched egg% mean ± st. deviation)	3.000 ± 1.000	2.000 ± 1.000	1.000 ± 0.577	0.333 ± 0.577	0.000 ± 0.000
Control (deltamethrin 1 μl/1 ml)			10.3 ± 4.231		
Control (ethyl alcohol 70%)			2.70 ± 2.354		

(*)Significant to control (ethyl alcohol) (P < 0.05).

*PG* *Pelargonium graveolens* oil, *PGN* *Pelargonium graveolens* nanoemulsion, *SO* sesame oil, *LS* linseed oil, *PGSO* *Pelargonium graveolens* oil + sesame oil.

### Larvicidal effect of PG formulations

The larvicidal effect of PG with mortality rates of 100 and 90.6% (P < 0.05) at the concentrations of 10 and 5%, respectively. PGN caused significant larval mortality with rates of 100, 100, 68.3, 58.3% (P < 0.05) at the concentrations of 10, 5, 2.5 and 1.25%, respectively. Also, PGSO revealed significant larval mortality (P < 0.05) at all the tested concentrations with 100% mortality at the concentrations of 5 and 10% and with 45% mortality at the concentration of 0.625% (Table 7). In contrary, SO and LS did not cause significant mortality against larvae at any of the tested concentrations. The LC_50_ of PG, PGN and PGSO were reached at the concentrations of 3.435, 1.688 and 0.944%, respectively. PGSO showed the lowest concentration needed to achieve the LC_50_ (0.944%) and the highest synergistic factor of 3.638 (> 1.00) (Table 8).

**Table 7:** Larvicidal effect of EOs (the ratio between oils in the binary mixture 1:1).

Concentrations%	10	5	2.50	1.25	0.625
PG (larval mortality%)	100.0 ± 0.000*	90.66 ± 1.154*	14.66 ± 0.577	8.333 ± 2.886	4.666 ± 0.577
PGN (larval mortality%)	100.0 ± 0.000*	100.0 ± 0.000*	68.33 ± 7.637*	58.33 ± 7.637*	8.333 ± 2.886
SO (larval mortality%)	19.00 ± 1.000	17.33 ± 2.081	15.66 ± 4.041	14.33 ± 4.041	7.666 ± 2.516
PGSO (larval mortality%)	100.0 ± 0.000*	100.0 ± 0.000*	91.66 ± 2.886*	76.66 ± 2.886*	45.00 ± 5.000*
LS (larval mortality%)	14.00 ± 4.041	10.33 ± 4.091	8.666 ± 2.886	4.333 ± 0.577	1.666 ± 0.577
Control (deltamethrin 1 μl/1 ml)			20.33 ± 0.577		
Control (phoxim 0.50 μl/1 ml)			100.0 ± 0.000		
Control (ethyl alcohol 70%)			0.000 ± 0.000		

*PG* *Pelargonium graveolens* oil, *PGN* *Pelargonium graveolens* nanoemulsion, *SO* sesame oil, *LS* linseed oil, *PGSO* *Pelargonium graveolens* oil + sesame oil.

(*)Significant to control (ethyl alcohol) (P < 0.05).

**Table 8:** LC_50_, LC_90_ and Synergistic factor of larvicidal activity of PG (the ratio between oils in the binary mixture 1:1).

Oil	LC_50_ %	LC_90_ %	Synergistic factor
PG	3.435	5.189	
PGN	1.688 *	3.004	
SO	21.56	41.35	
LS	23.56	44.44	
PGSO	0.944 *	1.966	3.638 (synergism)

*PG* *Pelargonium graveolens* oil, *PGN* *Pelargonium graveolens* nanoemulsion, *SO* sesame oil, *LS* linseed oil, *PGSO* *Pelargonium graveolens* oil + sesame oil.

### Field trial

**Figure 1:**
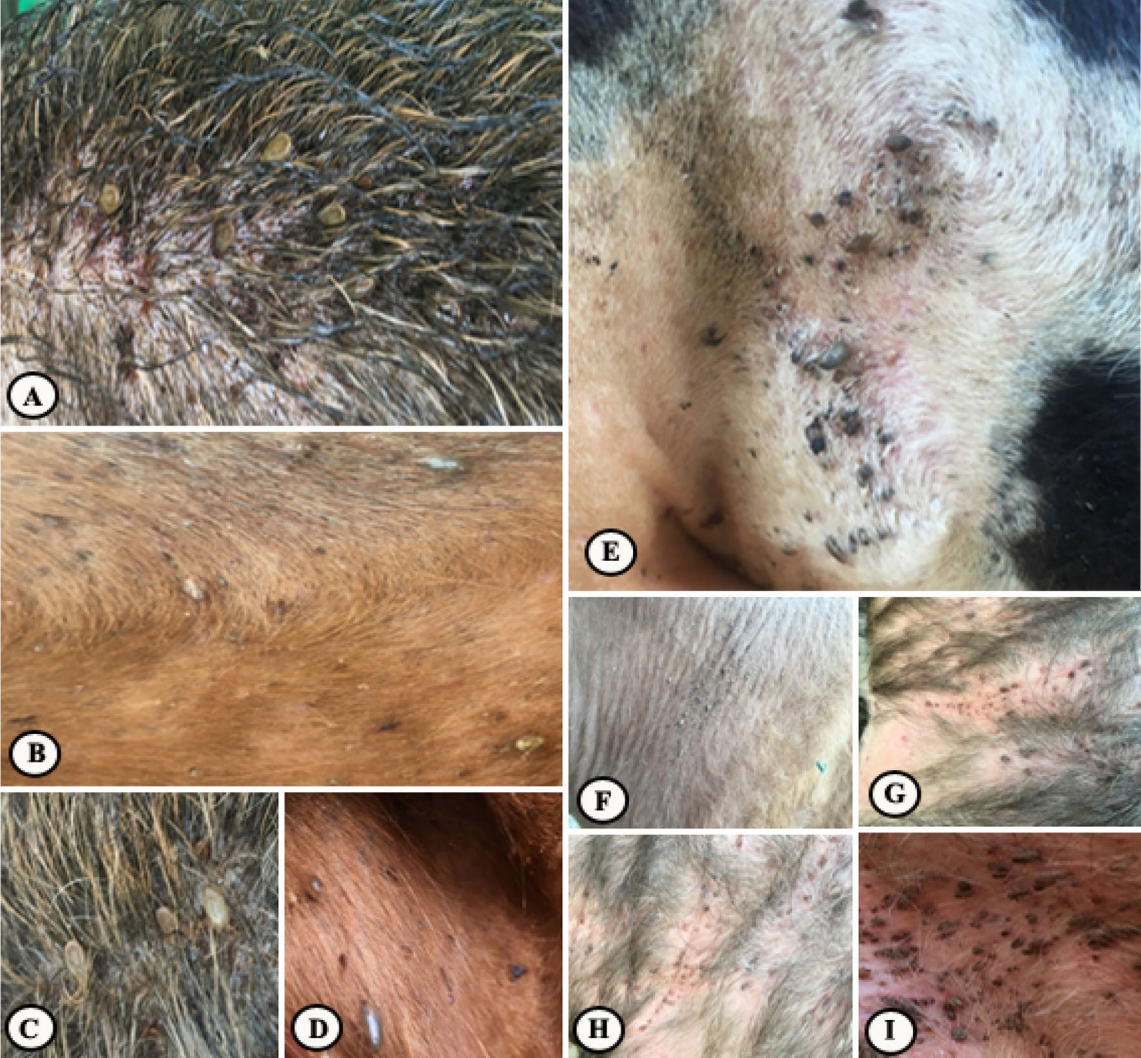
Natural heavy infestation of *Rhipicephalus annulatus* ticks at different parts of the cattle body (**A**–**C**) back, (**D**) leg, (**E**) shoulder, (**F**–**H**) neck, and (**I**) perineal region before the treatment by PGN.

**Figure 2:**
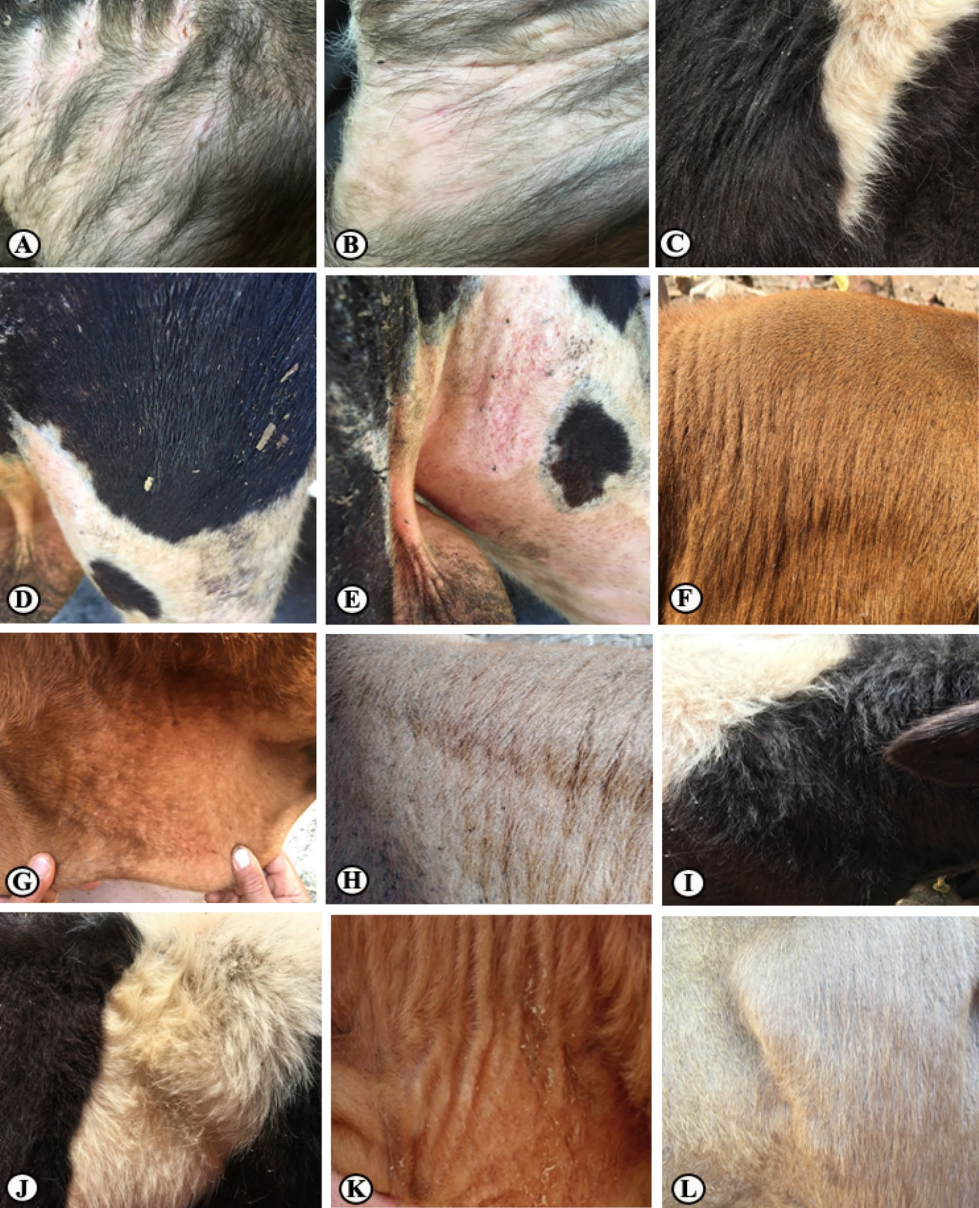
The natural infestation of *Rhipicephalus annulatus* ticks at different parts of the cattle on day 7 post-treatment with PGN showed a significant reduction in the tick counts at the different body parts.

**Figure 3:**
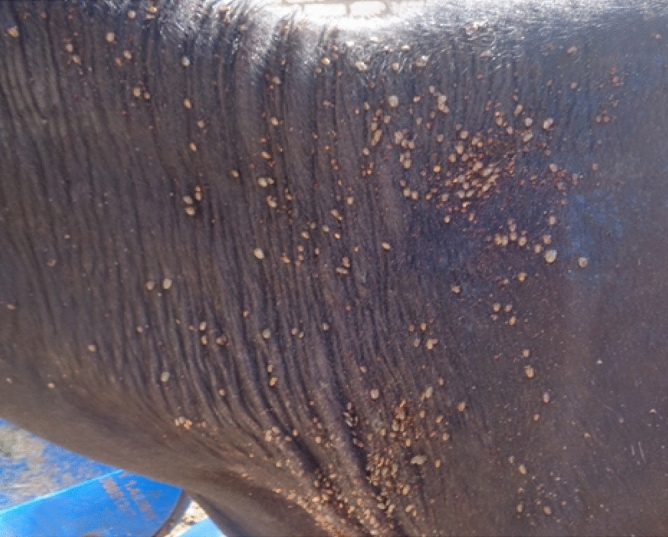
The neck and shoulder of cattle showed the natural infestation with *Rhipicephalus annulatus* ticks before the treatment with PGSO (geranium/sesame oil mixture).

**Figure 4:**
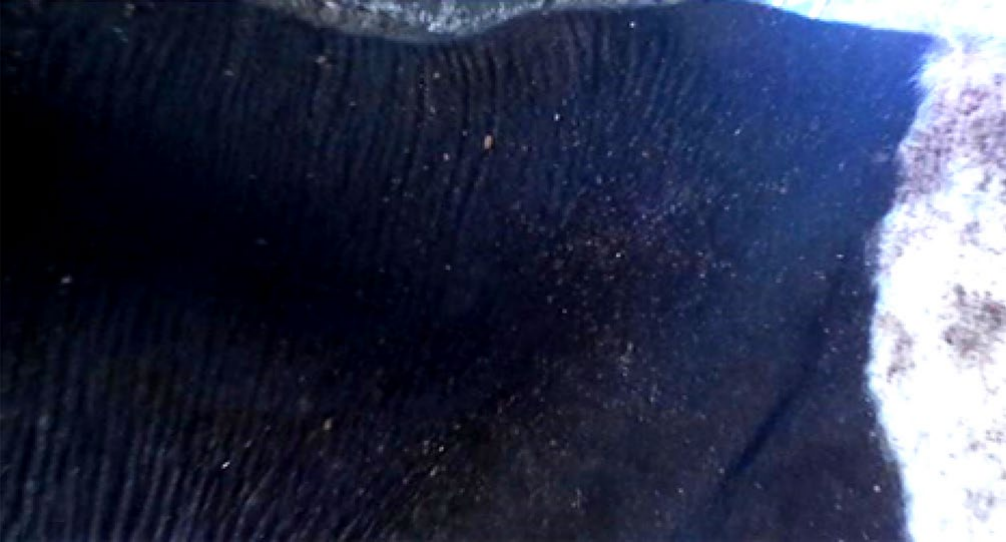
Cattle naturally infested with *Rhipicephalus annulatus* ticks showed a reduction in ticks count on day 7 post- treatment with PGSO.

**Figure 5:**
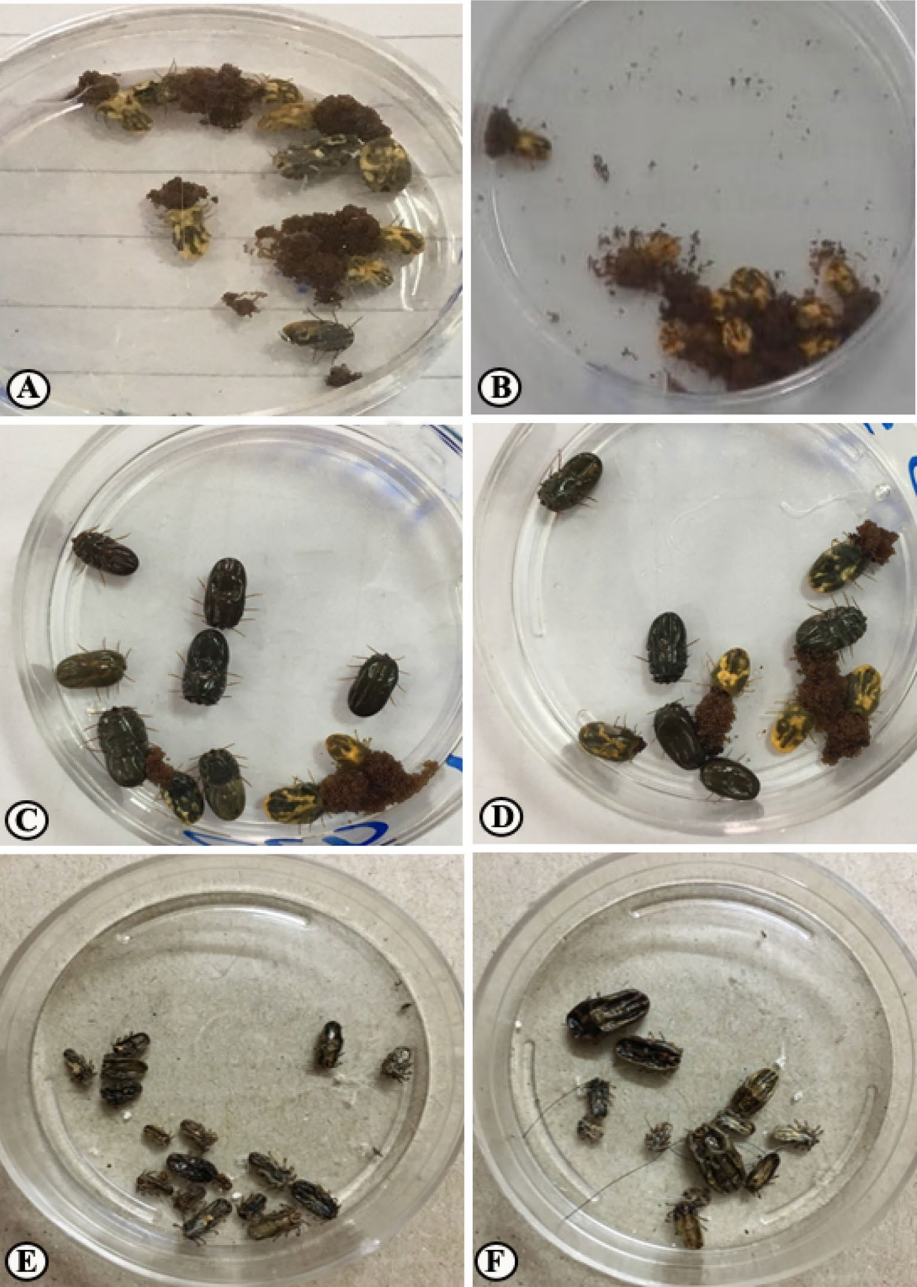
Collected *Rhipicephalus annulatus* ticks from treated cattle. (**A**,**B**) Treatment with deltamethrin showed normal egg deposition. (**C**,**D**) Treatment with PGSO showed only 3–4 ticks oviposited a few eggs. (**E**,**F**) Treatment with PGN showed dead ticks within the first hours and no egg deposition.

On day 3 post-application (PA), the ticks count was reduced to 50.59%, 68.69% on cattle treated with PGSO and PGN, respectively meanwhile cattle treated with deltamethrin showed only 21.26% reduction in the tick count. Eggs produced by ticks collected from animals treated with PGSO and deltamethrin hatched normally. Meanwhile, ticks collected from animals treated with PGN didn't oviposit any eggs (Figs. 1, 2, 3, 4, 5). On the day 7 PA, PGSO and PGN treated cattle showed 70.83% and 87.87% reduction in tick count, respectively. In contrast, the deltamethrin treated cattle showed only 28.26% reduction in the tick count. On the day 21 PA, the tick count were reduced to 74.83%, 87.97% and 29.88% on PGSO, PGN and deltamethrin treated animals, respectively (Table 9).

**Table 9:** Percent reduction of ticks by PGSO and PGN formulations in relation to deltamethrin in the control *R. annulatus* on naturally infested cattle.

Item group	No. of ticks on day zero PA	Reduction% of ticks number after 3 day	Reduction% of ticks number on day 7	Reduction% of ticks number on day 14	Reduction% of ticks number on day 21	Reproductive efficacy on day 7
Deltamethrin treated cattle	135.0 ± 5.000	21.15 ± 4.651	28.21 ± 1.875	27.91 ± 2.356	29.88 ± 1.852	47.73 ± 2.587*
Untreated control	136.0 ± 4.302	0000 ± 0000	0000 ± 0000	0000 ± 0000	0000 ± 0000	76.84 ± 3.949
PGSO treated cattle	138.3 ± 3.055	50.59 ± 5.443 *	70.83 ± 1.612 *	73.13 ± 2.312 *	74.83 ± 3.012 *	24.56 ± 4.382*
PGN treated cattle	133.0 ± 4.582	68.69 ± 4.542 *	87.87 ± 2.520 *	86.87 ± 1.930 *	87.97 ± 3.504 *	0000 ± 0000

*PGSO* *Pelargonium graveolens* oil + sesame oil, *PGN* *Pelargonium graveolens* oil nanoemulsion*.*

All the animals of this study in all groups were fed normally and did not display any abnormal clinical signs (normal appetite, no redness or itching, and no hair loss).

## Discussion

Tick control depends mainly on the use of synthetic chemical acaricides. However, the intensive use such chemicals led to the development of resistant tick populations. Therefore, it is necessary to look for alternative products to control ticks. Plant extracts represent a promising source of new acaricides due to their high safety and effectiveness. *Pelargonium graveolens* EO is widely used in manufacture of perfumes and the food industry as well as an it used as an antiseptic, an antioxidant, an antibacterial, an antifungal and treatment for cellulite reduction^19^.

The GC–MS analysis of PGEO revealed that the main components were citronellol (14.44%), geraniol (11.08%), and linalool (7.74%). In general, citronellol and geraniol are the important constituents that responsible for the acaricidal activity of EOs. The percentage of citronellol and geraniol in PG EOs differs according to many factors such as planting location and harvesting time^8^. This variation in chemical compositions also may be due to the chemotypic variation, soil characteristics, and age of the plant^20^. In addition, the geography, environmental conditions of the different regions, methods of extraction, and the plant part that used in extraction have an impact on the quality and quantity of extracted oil^21^. The best locations to produce geranium oil in Egypt are El-Qantara Sharq followed by El-Sadat City and finally Beni-Suef^8^.

The acaricidal activity of PG against *R.* (*B*.) *annulatus* was evaluated. The results showed that the PG caused 73.3 and 23.3% mortality at concentrations of 10, 5% respectively, meanwhile at the low concentrations PG revealed no significant (P > 0.05) acaricidal activity. Also, PG caused 100 and 90.6% larval mortality at the concentrations of 10 and 5% respectively, with no significant larval mortality at the concentrations of less than 5%. Also, PG at concentrations of 10 and 5% caused significant reduction in egg hatchability (P < 0.05). Similar acaricidal efficacy was reported to *Pelargonium roseum* and *Eucalyptus globulus* against *R. *(*B.*)* annulatus*. The extracts of these plants produced 98.3% and 37.6 adult mortality, respectively with 87.5 and 25% reduction in the egg laying, respectively, at the concentration of 5% on day 6 post-treatment^9^. The toxic effects of high concentrations of PGEO occurred through a neurotoxic pathway that causes hyperactivity followed by hyperexcitation then immobilization^22^. The acaricidal activities of *P. graveolens* were attributed to its main constituents which include geraniol, citronellol, and citronellyl formate^23^. Geranium is a natural source for l-quisqualic acid which useful for the production of botanically-based formulations for insect management^24^. l-Quisqualic acid acts as a reversible inhibitor for acetylcholinesterase similar to the action of linalool and terpene^25^. Geranium essential oil showed significant toxicity against the cowpea beetle, *Callosobruchus maculatus*^26^. The effect of *Pelargonium roseum* EO on the viability and ability of oviposition of *R.*(*B.*)* annulatus* might be due to the presence of geraniol (8.74%) which was proven to have powerful repellent activity for insect^10^. Also, *P. graveolens* was found to have a lethal effect against the house fly (*Musca domestica*)^27^.

SO exhibited powerful acaricide activity against the adult stages and weak activity against the other stages (eggs and larvae). The mode of action of SO on adult ticks is unknown. Generally, the toxic action of oils against ticks is more physical than chemical and often short-lived^28^.

To improve the acaricidal activity of PG, PG was used either in nanoemulsion form or in combination with food oils (sesame oil and linseed oil). PG nanoemulsion was prepared according to Nirmala et al.^29^ and characterized by zeta potential, zeta particle size and UV–Vis spectrophotometer to confirm that PG was reached to the nanostructure. PGN’s acaricidal activity was higher than PG with LC_50_ achieved at concentration of 5.60% and mortality rate of 96.6 and 30% in adult ticks at concentrations of 10 and 5%, respectively. These results indicate that toxicity of PG can be enhanced when it applied as a nanoemulsion. PGN when applied against tick larvae attained LC_50_ at concentration lower than that of PG. Also, PGN achieved greater ovicidal activity than PG. Dos Santos et al.^14^ reported significant acaricidal activities for nanostructured essential oils (nanoemulsion or nanocapsule) against *R.*(*B.*) *annulatus* ticks and they attributed these activities to the reduction of particle size, continued release of the oil, and increased the product stability. The small size of the droplet of EOs in nanoemulsion increases the amount of active molecule that contact to the biological target membranes and promotes their transfer through them^30^. In addition, nanoemulsion form of EOs improves their stability and minimizes the effective dose/application, prevents rapid evaporation and degradation, and reduces its toxicity to non-targeted organisms^31^. Adel et al.^32^ evaluated the efficacy of geranium EOs and geranium loaded on solid lipid nanoparticles (SLN) against the potato tuber larvae, *Phthorimaea operculella,* and they found that the SLN was more effective than geranium. Geranium nanoemulsions were confirmed to have antimicrobial efficacy against *Candida* spp.^33^. Skuhrovec et al.^34^ investigated the insecticidal efficacy of the EOs of *Rosmarinus officinalis* with *Cymbopogon citratus* and *Pelargonium graveolens* with *Thymus vulgaris* in combination and in nanoencapsulation forms against adults and larvae of *Oulema melanopus* and they found that the combination EOs and nanoencapsulation caused 100% mortality within 24 h. against larvae and adults. Also, *P. graveolens* infusion exhibited a relatively high anti-acetyl-cholinesterase activity and a considerable antimicrobial activity against *S. aureus*^35^.

The PGSO combinations at (1:1) ratio or at different ratios showed synergistic effects. The LC_50_ was reached at concentration of 1.91% with synergistic factor of 3.94 and 100% mortality was attained at the concentrations 10 and 5%. Moreover, the combinations of PGSO achieved better larvicidal activity than PG. Regarding the ovicidal activity, PGSO showed results similar to these of PG. The combination of PG and linseed oil revealed antagonistic effect with the LC_50_ reached at concentration of 15.45% with synergistic factor of 0.48 and with no significant mortality even at high concentrations. Benelli et al.^12^ tested many binary combinations between different essential oils and they found a high synergistic effect between *Pinus nigra* and *Aloysia citriodora* against larvae of *Culex quinquefasciatus*. They reported also a low effectiveness for the combination of *Hyssopus officinalis* and *Pelargonium graveolens*. Furthermore, Ramesh et al.^36^ found a synergistic effect for the combination of sesame oil with synthetic pyrethroids (deltamethrin) against *P. xylostella* and *Rhyzopertha dominica* in India. Similarly, Mesbah et al.^37^ recorded a synergistic effect for the combination of clove and sesame oils against the 4th larval instar of the cotton leaf-worm (*Spodoptera littoralis*). In addition, Soe et al.^17^ found similar synergism for sesame oil with clove oil against the pulse beetle (*Callosobruchus maculatus*).

A field trial to control ticks on naturally infested cattle was also carried out. The acaricidal effect of PGSO at a ratio of (9 PG:1 SO) against *R.*(*B.*)* annulatus* ticks on naturally infested cattle, revealed 74.83% reduction in tick count on day 21 PA. The PGN achieved the best result with 87.97% tick count reduction. This result is in agreement with those of Boito et al.^16^ as they found that the tea tree nanoparticles reduced the reproduction rate of *R. *(*B.*)* microplus* on dairy cattle to 34.5%. Also, Donsi et al.^13^ reported a significant reduction in the number of the adult ticks on infested dairy cattle and in female tick infertility after applications of pure cinnamon and nanocapsule form of cinnamon oil with a dose of nanocapsule 10 times lower than the dose of pure cinnamon oil. In the same way, Olivo et al.^38^ reported 79.3–91.2% efficacy for citronella oil in vitro and in vivo against cattle ticks.

The search for synergy among the essential oil constituents is a promising approach for increasing the acaricidal activity of natural compounds. The synergistic mixtures enable a defined level of effect with a dose of constituents lower than that of using the constituents alone. Thus, in the present study, mixing geranium oil with sesame oil could be a good strategy to improve the acaricidal activity and decrease the costs because of the lower price of the sesame than the geranium oil.

## Material and methods

### Preparation of the used materials

#### Preparation of plant oils

*Pelargonium graveolens* essential oil (PG), Sesame seed oil (SO) and Linseed oil (LS) were purchased from Trust scientific for natural products, Cairo, Egypt. These oils were dissolved in 70% ethanol to prepare 10, 5, 2.5, 1.25 and 0.625% (volume/volume). To investigate the synergism/antagonism action between PG oil and the other two food oils, binary mixtures from these oils were added at rate of (1:1) from the all five concentrations. To investigate which binary mixture is synergistic, PG with SO or with LS were added to each other from 1:1 till 9:1 and vice versa. The synergistic factor (SF) of essential oil + binary food oil was calculated according the following equation of Suwannayod et al.^39^.

$$\mathrm{SF}=\frac{\mathrm{LC}50\mathrm{ \,for \, the \,oil \,alone }}{\mathrm{LC}50\mathrm{ \,for \,the \,combination}}$$

SF > 1 indicates synergism; SF < 1 indicates antagonism.

#### GC–MS of PG oil

GC–MS analysis was carried out using a TRACE GC Ultra Gas Chromatograph (THERMO Scientific Corp., USA). The GC–MS system was equipped with a TR-5 MS column (30 m × 0.32 mm i.d., 0.25 μm film thickness). This analysis was done at Nawah Scientific Educational Research Center, Egypt (https://nawah-scientific.com/).

#### Preparation of PG nanoemulsion

PG nanoemulsion (PGN) consisted of three components; PGEO, Tween 80 (T80) and distilled water^29^. Creating the nanoemulsion formulation needed two steps. First, preparation of Oil/Water macroemulsion which was done by blending PGEO with T80 and water at one part oil to two T80 parts using a magnetic stirrer at a speed of 500 rpm for 10 min. The PGEO concentration was 10%. Second, the produced macroemulsion was subjected to ultrasonication with 750 W input power processor for 10 min (Branson Probe sonicator-Advanced model, 20 kHz) to form the respective nanoemulsion. The mild heat generated was regulated by placing the sample in a container filled with ice.

#### Characterization of PG nanoemulsion

Absorbance of the nanoemulsion was measured at 345 nm with the aid of UV–visible spectrophotometer (UV-2600, Shimadz, Japan). The zeta potential, droplet size distribution (d, nm) (analysis by volume) and polydispersity index (PDI) of nanoemulsions were measured using a zeta sizer apparatus (dynamic light scattering technique) (Nano-ZS90, Malvern, UK). Prior to the measurements, all the samples were diluted to 10% with deionized water to reduce multiple scattering effects.

### Acaricide activity of the prepared materials

#### Preparation of ticks, eggs and larvae

Fully engorged females of *R. *(*B.*) *annulatus* were collected from naturally infested cattle from El-Fayoum Governorate, Egypt (85 km south western to Cairo). The ticks were collected from cattle with history of recurrent tick infestation and did not receive any treatment for 1 month. The collected ticks were transported to the Parasitology Laboratory, Faculty of Veterinary Medicine, Beni-Suef University. The ticks species verification was completed according to Estrada-Peña^40^. The ticks were divided into petri dishes containing 10 ticks in each for Adult immersion bioassay. A second group of collected ticks were incubated in a BOD incubator and allowed to oviposit. Eggs were collected, mixed, and then allocated to test tubes in 50 mg lots. The allotted eggs were then incubated until hatching. The hatched larvae were used for larval packet test.

#### Adult immersion test (AIT)

The acaricidal effect of PG, PGN, SO, LS, PGSO, and PGLS was tested against adult ticks. This assay was done according to Drummond et al.^41^. Engorged female ticks were immersed in tubes containing 10 ml of different concentrations for 2 min, then dried and incubated at 26–28 °C and 80% relative humidity in petri dishes. Five replicates of 10 ticks for each concentration were conducted. The control ticks were treated by immersion in 70% ethanol for 2 min, another group was treated with deltamethrin (1 μl/ml) and a positive control was treated with 0.50 ml/l Phoxim. The results of these applications were evaluated by counting the number of dead adult ticks (DF), calculating the egg production index (EPI) and the hatchability percentage of eggs.

#### Ovicidal activity

50 mg of tick eggs were placed on a 7 cm diameter filter paper. One ml of each test concentration was applied to the eggs in a plastic mesh for 2 min. Then, eggs were dried on a filter paper, and incubated in glass tubes closed with cotton^41^. The control eggs were treated with 70% ethanol and 1 ml/l deltamethrin was used as a positive control. The eggs in the treated groups were incubated until hatching. Three replicates were used for each concentration.

#### Larvicidal activity

A modified larval packet technique (LPT) was used^42,43^. Approximately one hundred larvae were placed on filter papers (7 × 7 cm) using a fine tipped paint brush then 100 µl of the test solutions was added. The treated filter papers were then sealed to form packets. Control groups were treated with ethanol (70%) and 1 ml/l deltamethrin served as a positive control. For each concentration, 5 replicates were conducted. The treated packets were investigated after 24 h to record the mortality rates by counting live and dead larvae (motionless larvae were considered dead).

### Field trial

#### Animals

A field application was conducted on a total of 20 naturally infested cattle of native breed (18–24 month age, average weight 275–325 kg) in stalls at a small dairy cattle farm. The experiment was done during May 2021 (hot season). the selected cattle did not receive medications in the last month for any parasitic infection. Tick count was done on one side^44^. The animals were allocated in groups of an equal number of similar weight and tick count.

Based on the in vitro findings of binary mixtures between PG and SO, the best binary mixture was 9 parts of PG to 1 part of SO. Therefore, it was selected for the field trial. All animals were randomly divided and separated from each other by partitions into 4 equal groups (5 animals each) as following: PGSO, PGN, deltamethrin (recommended dose = 1 μl/ml distilled water), and untreated control groups. The treatment was done by spraying the animals one time. Each animal was sprayed with 400 ml of prepared PGSO solution dissolved in 70% ethyl alcohol. In the PGN group, each animal was sprayed with 400 ml as well. The animals in each group were examined for ticks on days 0, 3, 7, 14, and 21 post-application. From each group, 30 engorged female ticks of similar size were collected 72 h after treatment and incubated in the laboratory for determination of reproductive indices. The eggs deposited from the treated ticks were collected, weighed, and incubated for monitoring of egg hatchability. The treated animals were observed during the study for any abnormal signs (appetite, itching, or redness in the skin and hair loss). The efficacy was estimated by the percent of reduction in the count of the adult female ticks engorging on the treated cattle relative to the negative control animals^44^. The efficacy of treatment was calculated as follows: Efficacy (%) = 100 × (TC pre − TC post)/TC pre, where TC pre = is the mean number of live ticks on cattle before the treatment, TC post = the mean number of live ticks on cattle after the treatment^45^.

### Statistical analysis

Data of the different treatments was statistically analyzed using IBM SPSS for Windows, v.22 (IBM, Armonk, NY, USA). ANOVA was used to compare the different treatments and subsequent Duncan’s estimated the differences between means (α = 0.05). Lethal concentrations, causing 50% mortality were calculated with SPSS v.22.

### Ethical approval

The study was conducted under the roles of the ethical standards approved by Faculty of Veterinary Medicine, Beni-Suef University, Egypt, Approval number (021-172). All experiments were performed in accordance with relevant guidelines and regulations.

## Conclusion

The acaricide activity of PG can be improved when it applied either in form of PG nanoemlusion (PGN) or in combination with the sesame oil (PGSO). The application of these two forms on cattle naturally infested by ticks, achieved a 74.83% and 87.97% reduction in the count of deltamethrin-resistant ticks on day 21 PA for PGSO and PGN, respectively. However, further studies; especially in the field; were needed to fully understand the advantages of these formulations. Addition of SO to PG can improve its efficacy and reduce the cost of its application.

## Supplementary Information

Supplementary Video 1.

Supplementary Information 1.

## Data Availability

The datasets generated during and/or analyzed during the current study are available from the corresponding author on reasonable request.
