# Supplementary material for: Preparation of geranium oil formulations effective for control of phenotypic resistant cattle tick Rhipicephalus annulatus
Source: Sci Rep. 2022 Jul 8;12:11693. doi: 10.1038/s41598-022-14661-5 (PMC9270397; doi:10.1038/s41598-022-14661-5)
Supplement: Supplementary file 2 — Supplementary Information 1. [file 41598_2022_14661_MOESM2_ESM.docx]

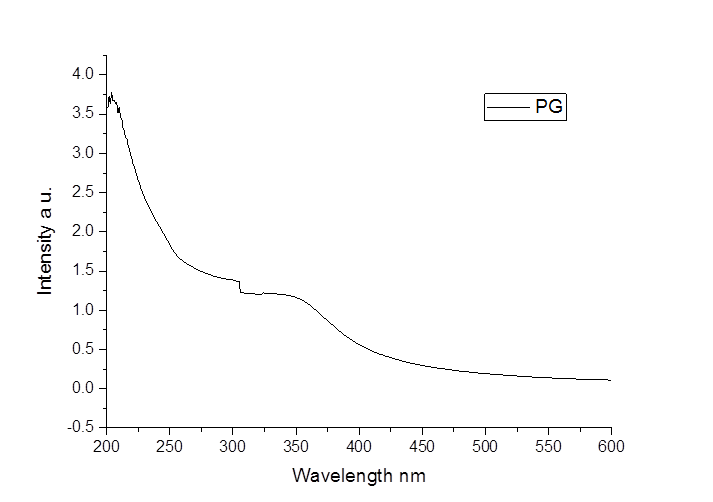


Figure 1: UV-vis spectrophotometer absorbance of PG

Figure 2: Size distribution of PG by intensity using Zeta apparatus

Figure 3: Zeta potential distribution of PG by Zeta apparatus
